# NHS-IL2 combined with radiotherapy: preclinical rationale and phase Ib trial results in metastatic non-small cell lung cancer following first-line chemotherapy

**DOI:** 10.1186/s12967-015-0397-0

**Published:** 2015-01-27

**Authors:** Michel M van den Heuvel, Marcel Verheij, Rogier Boshuizen, José Belderbos, Anne-Marie C Dingemans, Dirk De Ruysscher, Julien Laurent, Robert Tighe, John Haanen, Sonia Quaratino

**Affiliations:** Department of Medical Oncology, The Netherlands Cancer Institute/Antoni van Leeuwenhoek Hospital, Plesmanlaan 121 1066 CX, Amsterdam, The Netherlands; Department of Radiation Oncology, The Netherlands Cancer Institute/Antoni van Leeuwenhoek Ziekenhuis, Amsterdam, The Netherlands; Department of Pulmonology and GROW – School for Oncology and Developmental Biology, Maastricht University Medical Center, Maastricht, The Netherlands; Department of Radiation Oncology, Maastro, Maastricht, The Netherlands and University Hospitals Leuven/KU Leuven, Leuven, Belgium; Merck KGaA, Darmstadt, Germany; EMD Serono, Billerica, MA, USA subsidiary of Merck KGaA, Darmstadt, Germany; Present address: Novartis Pharma, Basel, Switzerland

**Keywords:** Lung cancer, NHS-IL2, Immunotherapy, Radiotherapy, Phase Ib

## Abstract

**Background:**

NHS-IL2 (selectikine, EMD 521873, MSB0010445) consists of human NHS76 (antibody specific for necrotic DNA) fused to genetically modified human interleukin 2 (IL-2) and selectively activates the high-affinity IL-2 receptor. Based on an evolving investigational concept to prime the tumor microenvironment with ionizing radiation prior to initiating immunotherapy, 2 related studies were conducted and are reported here. The first, a preclinical study, tests the systemic effect of the immunocytokine NHS-IL2 and radiotherapy in a lung carcinoma animal model; the second, a phase Ib trial in patients with metastatic non-small cell lung carcinoma (NSCLC), was designed to determine the safety and tolerability of NHS-IL2 in combination with radiotherapy directly following first-line palliative chemotherapy.

**Methods:**

Tumor-bearing C57Bl/6 mice were treated with NHS-IL2 alone (5 mg/kg; days 7–9), fractionated radiotherapy (3.6 Gy; days 0–4) plus cisplatin (4 mg/kg; day 0), or the triple combination. Metastatic NSCLC patients who achieved disease control with first-line palliative chemotherapy were enrolled in the phase Ib trial. Patients received local irradiation (5x 4 Gy) of a single pulmonary nodule. Dose-escalated NHS-IL2 was administered as 1-h intravenous infusion on 3 consecutive days every 3 weeks.

**Results:**

NHS-IL2 plus radiotherapy induced immune response activation and complete tumor growth regressions in 80%–100% of mice. In patients with metastatic NSCLC treated with NHS-IL2 (3, 3, and 7 patients in the 0.15-mg/kg, 0.30-mg/kg, and 0.45-mg/kg cohorts, respectively), maximum tolerated dose was not reached. Most frequently reported adverse events were fatigue, anorexia, and rash. Transient increases in leukocyte subsets were observed. In 3 patients, thyroid gland dysfunction occurred. No objective responses were reported; long-term survival was observed in 2 patients, including 1 patient with long-term tumor control.

**Conclusions:**

Combining NHS-IL2 with radiotherapy achieved synergistic antitumor activity in preclinical studies, supporting the use in lung cancer patients. This combination was well tolerated and 2 of 13 patients achieved long-term survival.

**Trial registration:**

ClinicalTrials.gov NCT00879866

**Electronic supplementary material:**

The online version of this article (doi:10.1186/s12967-015-0397-0) contains supplementary material, which is available to authorized users.

## Background

Metastatic non-small cell lung cancer (NSCLC) has a dismal prognosis, with a median overall survival (OS) of about 1 year in patients treated with standard platinum-based chemotherapy. Targeting agents such as epidermal growth factor receptor (EGFR) tyrosine-kinase inhibitors (TKIs) or crizotinib lead to progression-free survival (PFS) that lasts for an average of 8–12 months [1,2]. Several trials investigating cancer vaccines and immune checkpoint blockade in patients with metastatic NSCLC showed promising results in terms of tumor regression and survival response [3-5], and a series of phase III trials are currently ongoing [6]. Active immunotherapy has the potential to induce ongoing lasting therapeutic benefit for this indication.

Experience with interleukin 2 (IL-2) in advanced-stage NSCLC is limited. Uncontrolled phase II studies suggested a favorable survival for patients receiving IL-2 comparable to chemotherapy [7]. A phase II, nonrandomized, pilot study in patients with advanced NSCLC demonstrated enhanced efficacy of a TKI, gefitinib, in combination with IL-2 compared with gefitinib alone [8]. In a controlled phase III trial in early stage disease after resection, patients with NSCLC received IL-2 and lymphokine-activated killer cells following adjuvant chemotherapy or radiotherapy in the experimental arm. Patients in the control arm received chemotherapy or radiotherapy only. A statistically significant difference in the 5-year survival rate was reached (54% vs. 33%; *P* < 0.001) [9]; however, IL-2 given at high doses can cause severe toxicity. Therefore, no other large, controlled studies have been performed in advanced-stage NSCLC.

NHS-IL2 (selectikine, EMD 521873, MSB0010445) is a novel immunocytokine comprising a human tumor necrosis-targeting antibody (NHS76) that binds to exposed DNA in necrotic regions of tumors, fused to genetically modified IL-2 designed to decrease vascular toxicity by signaling through the high-affinity IL-2 receptor [10,11]. A phase I trial in solid tumors has shown the safety and tolerability of NHS-IL2 as monotherapy in humans, reaching a maximum tolerated dose (MTD) at 0.6 mg/kg [11]. The aim of this phase Ib trial was to combine NHS-IL2 with radiotherapy. The traditional palliative role of radiotherapy in metastatic disease is evolving into an investigational concept of an initiator for immunotherapy [12,13]. Thus, it was hypothesized that the administration of NHS-IL2 following local tumor irradiation would provide systemic control of tumor growth [12,14-18]. The immunogenic cell death induced by radiation [12] would increase tumor-targeting of NHS-IL2, leading to an enhanced activation of tumor-specific cytotoxic T-lymphocytes (CTLs) and other immune effector cells via the low-toxicity formulation of IL-2. Experimental data from several cancer models have shown that some of the effects of ionizing radiation are recognized as contributing to systemic antitumor immunity, the so-called abscopal effect [13]. Therefore, this combined treatment could target more than one of the hallmarks of cancer by modifying the tumor microenvironment and promoting inflammation, and also by directly expanding tumor-antagonizing CTLs and natural killer (NK) cells [19]. The developmental concept is to introduce a new treatment modality of “immunostimulating” local irradiation followed by IL-2–based immunocytokine therapy in patients with metastatic NSCLC who have at least stable disease after first-line platinum-based chemotherapy. Based on substantial evidence that the immune system and inflammatory processes play a role in the development of NSCLC, despite it being a malignancy with low immunogenicity, a combination approach of radiotherapy plus immunotherapy could potentially lead to an active and specific immune response [6]. In metastatic melanoma or renal cell carcinoma, a similar concept of stereotactic body radiation therapy followed by high-dose IL-2 has shown promising clinical responses in 8 out of 12 patients [20].

Here we report on a composite of a preclinical animal study and a phase Ib trial in metastatic NSCLC to test the systemic effect of the immunocytokine NHS-IL2 and radiotherapy in an animal model, and determine safety and tolerability of NHS-IL2 in combination with radiotherapy in metastatic NSCLC directly following first-line palliative chemotherapy.

## Methods

### Experimental model

A murine Lewis lung carcinoma (LLC) cell line (gift from Children’s Hospital, Boston, MA) was maintained in Dulbecco’s Modified Eagle’s Medium (DMEM), supplemented with 10% heat-inactivated fetal bovine serum, L-glutamine, and penicillin/streptomycin (Gibco®, Carlsbad, CA, USA) and incubated at 37°C and 7% CO_2_. The cell line was obtained in 1996 and a master cell bank was frozen down at that time. A second cell bank, derived from the first, was frozen down in 2004, and this bank was used for the studies described in the manuscript. In 2007, prior to being used for the studies described in the manuscript, the cell line underwent polymerase chain reaction (PCR) testing for mycoplasma and common pathogenic murine viruses; the results of the tests were negative. Testing was performed by the University of Missouri RADIL Laboratory. The animal studies were performed at EMD Serono, Billerica, MA, USA, and animal care was in compliance with the animal welfare guidelines. Eight-week-old female C57Bl/6 mice were purchased from Charles River Laboratories, Wilmington, MA, and housed in standard cages with isolator tops. Groups of 10 mice were inoculated into the right quadriceps muscle with 1x10^5^ LLC cells. On day 0, 7 days after inoculation, mice were sorted into treatment groups with mean tumor volumes of ~110 mm^3^. The mice were treated with NHS-IL2 alone (5 mg/kg on days 7–9), fractionated local radiotherapy alone (3.6 Gy on days 0–4), cisplatin alone (4 mg/kg on day 0), radiotherapy plus cisplatin, NHS-IL2 plus cisplatin, NHS-IL2 plus radiation, or the triple treatment combination. Tumor-bearing legs were irradiated by timed exposure to a 137Cs source (GammaCell 40 Exactor, MDS Nordion, Ottawa, ON, Canada); a lead collimator device was used to localize delivery (MDS Nordion, Ottawa, ON, Canada). Tumors were measured twice weekly throughout the study using digital calipers, and tumor volume was calculated as: Volume = (length) × (width) × (height) × 0.5236. Mice were euthanized when tumor volume exceeded 2.5cm^3^.

### Clinical study design and patient population

The open-label, phase Ib trial (NCT00879866) used the classical “3 + 3 rule” design [21] to test NHS-IL2 at the escalating doses of 0.15 mg/kg, 0.30 mg/kg, or 0.45 mg/kg administered intravenously (i.v.) in combination with local irradiation (5x 4 Gy). After institutional review board approval, written informed consent was obtained and patients were enrolled at 3 clinical sites. Patients were deemed eligible in the presence of histologically or cytologically confirmed metastatic NSCLC, with disease control after 4–6 cycles of first-line platinum-based chemotherapy. No prior treatment with TKIs was allowed. Other important inclusion criteria were Eastern Cooperative Oncology Group (ECOG) performance status 0 or 1 and the presence of a pulmonary primary tumor or ≥1 NSCLC metastasis ≥1 cm in diameter and eligible for local radiotherapy with 5 fractions of 4 Gy. Main exclusion criteria were the requirement for immunosuppressive treatment, with the exception of inhalation corticosteroids or low-dose systemic corticosteroids (prednisone equivalent dose ≤10 mg/day); systemic autoimmune disease (e.g., lupus erythematosus, rheumatoid arthritis); active infections (including human immunodeficiency virus, hepatitis B and C, tuberculosis); and known brain metastases.

### Radiotherapy

Prior to the first NHS-IL2 treatment cycle all patients received a local irradiation dose of 20 Gy administered in fractions of 4 Gy on 5 consecutive days (day –7 to day –3, normally from Monday to Friday). Radiation was administered to the primary tumor or a pulmonary metastasis. Radiation technique and dose specifications were performed according to international guidelines [22]. In patients with multiple metastatic lesions the “dominant lesion”, defined as the lung metastasis with the largest diameter, was irradiated.

### Administration of NHS-IL2

Patients received NHS-IL2 at escalating doses of 0.15 mg/kg, 0.30 mg/kg or 0.45 mg/kg, as once-daily 1-hour i.v. infusion for 3 consecutive days, followed by an 18-day break (i.e., 21-day cycle). The first cycle started after a treatment-free interval of 2 days (weekend) following the end of radiation; the first dose in this cycle was given on a Monday (day 1). If this cycle was tolerated without occurrence of toxicities requiring treatment discontinuation, NHS-IL2 maintenance cycles were given every 21 days until the occurrence of intolerable side effects or clinically and radiologically relevant progression of disease. Recruitment of patients was to be stopped when the MTD was reached or when a total of 6 patients received 0.45 mg/kg NHS-IL2.

### Safety monitoring

Safety evaluations (clinical examination and laboratory assessments) were performed for all patients at baseline and at 6-week intervals. The severity of adverse events (AEs) was graded according to the National Cancer Institute Common Terminology Criteria for Adverse Events (NCI CTCAE), version 3.0 [23]. Safety data were discussed by an independent safety monitoring committee following each dose cohort and prior to the opening of the next dose level.

### Pharmacokinetics

For the quantitative NHS-IL2 detection, serum samples were analyzed using a specific enzyme-linked immunosorbent assay (ELISA), developed and validated at BURECO AG, a GLP/GMP certified laboratory, Reinach, Switzerland. Briefly, the NHS-IL2 assay employed a 2-step ELISA procedure for quantitative detection. NeutrAvidin-precoated microtiter plates were incubated with biotinylated ssDNA (17mer nucleotide). Calibration samples, quality control samples, and unknown samples were pipetted into defined wells. After incubation and washing away any unbound material, a primary monoclonal antibody (mAb) against IL2 was added. After a further incubation and washing step, a secondary horseradish peroxidase-linked Ab against immunoglobulin G was added. A wash step followed to remove any unbound antibody-enzyme reagent. Subsequently, 3,3′5,5′-tetramethylbenzidine (TMB), a chromogenic substrate solution, was added. The enzyme bound to the secondary Ab oxidized the substrate and produced a chromogen. The reaction was stopped by addition of sulfuric acid. The amount of colored product was directly proportional to the concentration of NHS-IL2 and was quantified in an absorbance plate reader at 450 nm (reference at 630 nm). Data analysis was performed with SoftMax Pro software (Molecular Devices, Sunnyvale, CA, USA). Pharmacokinetic analysis was performed at the following time points: cycle 1(days 1, 2, and 3) at 0 hour (pre-dose), 1 hour after start of infusion (end of infusion), and 4 to 8 hours after start of infusion; and at days 4 and 8. Further pharmacokinetic analysis was performed at cycle 2 and 4 (days 1, 2, and 3) at 0 hour (pre-dose), and 1 hour after start of infusion (end of infusion), and then at day 8.

### Immune response evaluation

Blood samples from patients were collected in lithium-heparin tubes (Vacutainer® blood collection tubes, BD, Franklin Lakes, NJ, USA ) before the start of irradiation (day –7) and on day 1 (before start of infusion of NHS-IL2) and on day 8 post-infusion of cycle 1; then subsequently on days 1 and 8 of cycles 2 to 4 and every fourth cycle. Besides white blood cell differential count, an immunomonitoring (flow cytometry analysis) was performed on fresh blood according to the following kinetic: before irradiation (day –7), on day 1, and day 8 of cycle 1 and 4. Briefly, after lysis of erythrocytes, cells were stained with the following mAbs: fluorescein isothiocyanate 1 (FITC)-conjugated anti-CD3, anti-Ki67; R-Phycoerythrin (PE)-conjugated anti-CD197; PE-Cy7-conjugated anti-CD4, anti-CD56; allophycocyanin (APC)-conjugated anti-CD45RA; APC-H7–conjugated anti-CD8; Pacific blue-conjugated anti-human leukocyte antigen D-related (HLA-DR), anti-CD16 and Amcyan-conjugated anti-CD3, according to manufacturer instruction. Isotype controls were used for the analysis. All antibodies were purchased from BD Pharmingen (BD, Franklin Lakes, NJ, USA). Data were acquired using FACSCanto II flow cytometer (BD, Franklin Lakes, NJ, USA), and analyzed with FACSDiva software v6.0 (BD, Franklin Lakes, NJ, USA) to assess the different lymphocyte subsets. In addition, whole blood was also collected in Vacutainer SST tubes (BD, Franklin Lakes, NJ, USA) on days –7 (before the start of radiotherapy), and on day 1 and 3 of cycle 1. Serum levels of soluble IL-2 receptor alpha (sIL-2Ralpha), interferon-gamma–induced protein (IP)-10 (CXCL10) (R&D systems, Minneapolis, USA), and neopterin (IBL International GmbH, Hamburg, Germany) were measured by ELISA according to the manufacturer’s instructions. Circulating levels of cytokines, which peak at a different time with respect to therapeutic administration, were not measured, nor were tumor biopsy data collected in this study.

### Statistics approach

The primary endpoint for this trial was the incidence of dose-limiting toxicity (DLT) occurring during the first cycle of administration of any dose of NHS-IL2 following radiotherapy. DLT was defined by any grade ≥3 toxicity during the first cycle of treatment (i.e., day 1–21) assessed as related to trial treatment by the investigator and/or sponsor and confirmed by the safety monitoring committee to be relevant to the combination treatment. Secondary endpoints included the incidence of treatment-emergent AEs, and monitoring of laboratory parameters and vital signs to be descriptively assessed on the safety populations (ie, all patients who had received ≥1 dose of radiation or NHS-IL2). Clinical outcome was explored by means of descriptive statistics on the endpoints of best overall response according to Response Evaluation Criteria in Solid Tumors (RECIST) version 1.0 [24], and PFS and OS, both analyzed with the Kaplan-Meier method and measured from the start of trial-based treatment. Response endpoints were analyzed on the efficacy analysis set (i.e., all patients who had received ≥1 infusion of NHS-IL2 and had ≥1 post-baseline assessment). Patients with no measurable disease but with clinically reported signs of disease progression were discontinued from trial medication and were included in the efficacy analysis set.

## Results

### Experimental model

**Figure 1:**
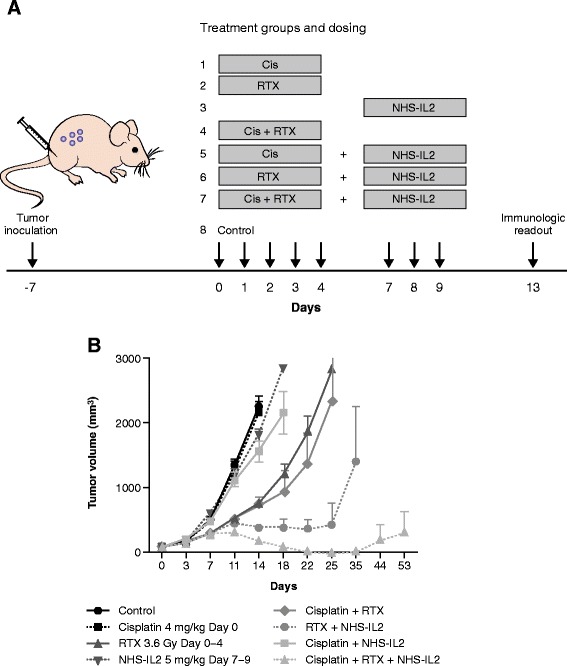
**Mouse model (LLC in C57/Bl6) study design (A) and observed tumor growth inhibition (B).** Mice received the indicated treatments when tumors had reached volume of 81 mm^3^ to 137 mm^3^ 7 days after implantation. Cisplatin 4 mg/kg was administered only on day 0. The dose of radiotherapy was 3.6 Gy and the dose of NHS-IL2 was 5 mg/kg. Each treatment group comprised 10 mice through day 14, and 6 mice thereafter; 4 mice from each group, selected around the mean, were sacrificed on day 14 for mechanistic endpoints. The experiment was repeated 3 times with similar results. Cis, cisplatin; RTX, radiotherapy.

We have previously demonstrated that NHS-IL2 is effective in a lung metastasis model and shown that the efficacy is primarily dependent on CD8+ T cells, and to a lesser extent on NK cells [10]. To test the hypothesis of whether radiotherapy affects immunity against tumor cells in metastatic disease, low-dose fractionated local radiation was combined with NHS-IL2 treatment in a syngeneic LLC tumor model. Seven days after implantation, the tumor reached a volume of 110 mm^3^ and 10 mice per group were treated with saline (i.e., control), NHS-IL2 alone, radiotherapy alone, cisplatin alone, NHS-IL2 plus cisplatin, NHS-IL2 plus radiation, radiation plus cisplatin, or the triple combination of cisplatin, radiation, and NHS-IL2 (study design see Figure 1A). NHS-IL2 alone did not significantly inhibit tumor growth, nor did cisplatin alone or cisplatin plus NHS-IL2. Radiotherapy alone delayed tumor growth only temporarily. Addition of cisplatin to either NHS-IL2 or radiotherapy did not achieve any significant improvement in tumor growth control compared to NHS-IL2 alone or radiotherapy alone. However, the combination of cisplatin plus radiotherapy with NHS-IL2 resulted in marked tumor reduction and delayed outgrowth that was statistically superior to all other groups (*P* < 0.001 by Bonferroni’s post-test following 2-way repeated-measures analysis of variance [ANOVA] on study day 14 [last day on which all study groups were complete]) (Figure 1B). At day 53 of the study, 5 of 6 mice remaining in the triple combination group had achieved complete regression. These data were reproduced in 2 experiments with similar designs (data not shown). The addition of cisplatin to the combination of radiotherapy and NHS-IL2 (ie, triple combination) contributed mainly to immune activation and tumor growth control. Following treatment, gene expression in tumors was determined by quantitative PCR, cellular changes in the periphery were monitored by fluorescence-activated cell sorting (FACS), and tumor-infiltrating lymphocytes assessed by immunohistochemistry (IHC) to comprehensively elucidate the mechanistic pathways involved in the synergistic antitumor effects of these combination therapies (data not shown; manuscript in preparation). Additional file 1: Figure S1 shows upregulated expression of genes associated with effector T cells (CD3, CD4, CD8, CD25) and cytotoxic immune cells (Fas ligand, granzyme B, perforin 1) within tumors treated with the triple combination, indicating an increased infiltration of activated T cells. This was further confirmed by FACS and IHC-based analysis of tumors, supporting the previously described immune-potentiating effects that radiation has on tumors (data not shown).

### Clinical study

**Figure 2:**
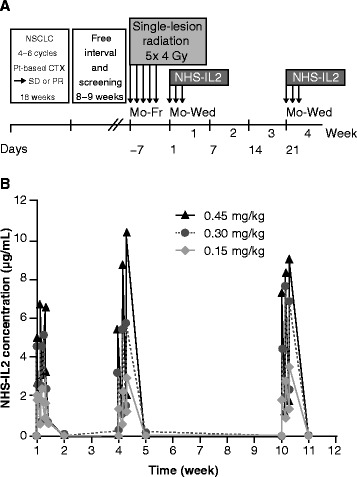
**Clinical trial design (A) and observed NHS-IL2 serum concentrations (B)**. Patients with metastatic NSCLC after first-line platinum-based therapy (4–6 cycles) were treated with radiotherapy followed by NHS-IL2 at escalating doses of 0.15 mg/kg, 0.30 mg/kg, or 0.45 mg/kg. Mean serum concentration of NHS-IL2 is depicted by dose level during the first, second, and fourth treatment cycles. CTX, chemotherapy; NSCLC, non-small cell lung cancer; PR, partial response; Pt, platinum; SD, stable disease.

Based on the promising preclinical study results, the EMR62235-002 phase Ib trial (NCT00879866) was initiated. Between April 2009 and August 2010 a total of 15 patients with NSCLC stage IV disease who achieved at least stable disease after first-line platinum-based therapy provided informed consent. Two patients showed progressive disease during screening and were thus not enrolled into the trial, leading to a total of 13 patients treated, as part of the safety analysis set (3 patients each in the 0.15-mg/kg and 0.30-mg/kg cohort, and 7 in the 0.45-mg/kg cohort). Baseline characteristics for this population are reported in Table 1. The overall median time from metastasis diagnosis to study entry was about 4.3 months, in which the patients received 4–6 courses of platinum doublets (Table 1). The 13 patients received 5× 4 Gy radiotherapy followed by NHS-IL2 over 3 consecutive days in a 21-day treatment cycle repeated until progression of disease or unacceptable toxicity (study design see Figure 2A). Twelve patients were evaluable to assess DLTs and antitumor activity, whereas the thirteenth patient had a major protocol deviation and was replaced. This patient received incorrect dosing (0.30 mg/kg instead of 0.45 mg/kg) at cycle 1 day 1, and was excluded from the DLT analysis set. Thirteen patients received a median of 3 completed cycles NHS-IL2 (mean number of completed cycles: 3.9 [standard deviation 3.9], range 1–12). In fact, 1 patient has received over 20 treatment courses.

**Table 1:** **Baseline characteristics – NSCLC patient population treated with radiotherapy and NHS-IL2 directly following first-line chemotherapy**

	**Overall (n = 13)**
**Age (years) mean ± SD**	59.7 ± 8.9
**Male/Female, n**	9/4
**ECOG performance status, n (%)**	
0	11 (85)
1	2 (15)
**Histology, n (%)**	
Adenocarcinoma	7 (54)
Squamous cell carcinoma	1 (8)
Undifferentiated carcinoma	2 (15)
Large cell carcinoma:	3 (23)
**Smoking status, n (%)**	
Current	2 (15)
Former	9 (69)
Never	2 (15)
**Previous chemotherapy (4–6 cycles), n**	
Cisplatin/carboplatin + gemcitabine	10
Carboplatin/cisplatin + pemetrexed (n = 2) or docetaxel (n = 1)	3

ECOG, Eastern Cooperative Oncology Group; NSCLC, non-small cell lung cancer; SD, standard deviation.

### Safety

The treatment was generally well tolerated (Table 2). Most frequently reported AEs were fatigue, anorexia, and rash (Table 2), often presenting with erythema with mild facial and periorbital swelling, and mild urticaria on the upper part of the thorax, most prominent from day 4 to about day 7 and followed by dry skin and exfoliation. These AEs were always followed by complete recovery within the second week and tended to recur after subsequent cycles, but were very manageable. There were only 3 grade 3 AEs considered by the investigator as related to NHS-IL2 (Table 2). One patient in the 0.30-mg/kg cohort developed grade 3 normochromic normocytic anemia, while grade 3 transitory lymphopenia was observed in 1 subject at 0.45 mg/kg. One DLT was reported also at the highest dose of NHS-IL2; the patient experienced grade 3 pericarditis directly following the first course, explained by rapidly progressive disease, as confirmed by postmortem examination.

**Table 2:** **Reported AEs (all grades)** ^**a**^ **during treatment with NHS-IL2 in advanced-stage NSCLC patients, deemed treatment related**

		**0.15 mg/kg (n = 3)**	**0.30 mg/kg (n = 3)**	**0.45 mg/kg (n = 7)**	**Overall (n = 13)**
**General disorders, n (%)**	Anorexia	3 (100.0)	-	2 (28.6)	5 (38.5)
	Fatigue	3 (100.0)	2 (66.7)	2 (28.6)	7 (53.8)
	Fever	2 (66.7)	2 (66.7)	-	4 (30.8)
	Flu-like illness	-	-	3 (42.9)	3 (23.1)
	Malaise	-	-	2 (28.6)	2 (15.4)
**Blood and lymphatic system disorders, n (%)**	Lymphopenia^b^	-	-	1 (14.3)	1 (7.7)
	Normochromic normocytic anemia^b^	-	1 (33.3)	-	1 (7.7)
**Gastrointestinal symptoms, n (%)**	Abdominal pain upper	2 (66.7)	-	-	2 (15.4)
	Diarrhea	-	1 (33.3)	1 (14.3)	2 (15.4)
	Eructation	2 (66.7)	-	-	2 (15.4)
	Nausea	2 (66.7)	-	1 (14.3)	3 (23.1)
	Stomatitis	1 (33.3)	-	1 (14.3)	2 (15.4)
**Respiratory symptoms, n (%)**	Dyspnea	1 (33.3)	1 (33.3)	2 (28.6)^b^	4 (30.8)
	Productive cough	1 (33.3)	-	1 (14.3)	2 (15.4)
**Skin disorders, n (%)**	Dry skin	-	2 (66.7)	2 (28.6)	4 (30.8)
	Pruritus	1 (33.3)	1 (33.3)	1 (14.3)	3 (23.1)
	Facial/periorbital edema^c^	-	1 (33.3)	1 (14.3)	2 (15.4)
	Peripheral edema	-	1 (33.3)	2 (28.6)	3 (23.1)
	Rash (any)	1 (33.3)	2 (66.7)	3 (42.9)	6 (46.2)
	Erythema	1 (33.3)	1 (33.3)	2 (28.6)	4 (30.8)
	Rash maculopapular	1 (33.3)	1 (33.3)	1 (14.3)	3 (23.1)
	Exfoliative rash	-	1 (33.3)	1 (14.3)	2 (15.4)
**Other, n (%)**	Atrial fibrillation	-	-	2 (28.6)	2 (15.4)
	Dry eye	-	1 (33.3)	1 (14.3)	2 (15.4)
	Hyperthyroidism	-	2 (66.7)	1 (14.3)	3 (23.1)
	Pericarditis malignant^b^	-	-	1 (14.3)	1 (7.7)

^a^AEs were graded according to NCI CTCAE v3.0 and all AEs except where noted were grade 1/2.

^b^Grade 3-4 AEs occurring in patients during treatment with NHS-IL2; 1 of the 2 patients who reported dyspnea experienced dyspnea grade 4.

^c^The facial edema and periorbital edema are in the same patients.

AE, adverse event; NCI-CTCAE, National Cancer Institute Common Terminology Criteria for AEs; NSCLC, non-small cell lung cancer.

In addition to the mild transient facial edema, the only classic IL-2 immune-related AE was thyroiditis. Three out of 12 patients developed hyperthyroidism grade 1/2 during treatment, with free thyroxin levels >100 pmol/dL after the second or third cycle. High levels of thyroglobulin accompanied the thyroiditis episodes. No autoantibodies against thyroid-stimulating hormone or thyroglobulin were detected in any of these patients during or after treatment. One patient had hyperthyroidism classified as a serious AE, developed symptomatic tachycardia and had to be treated with propranolol. This patient recovered completely after the treatment was stopped because of disease progression. Two patients, in whom treatment was continued, developed subsequent hypothyroidism that required thyroid hormone therapy supplements.

### Pharmacokinetics

Mean peak concentrations of NHS-IL2 in serum measured at the end of the 1-hour infusion on days 1–3 of cycle 1, 2, and 4 increased with dose in an almost directly proportional manner (Figure 2B). Within each cycle the peak concentrations continuously increased during the 3 days of administration until steady state was reached with the third administration. After the third administration concentrations declined steadily and reached pre-dose levels in week 2 of each cycle. No accumulation in exposure over time was observed at any NHS-IL2 dose level.

### Induction of immune response

**Figure 3:**
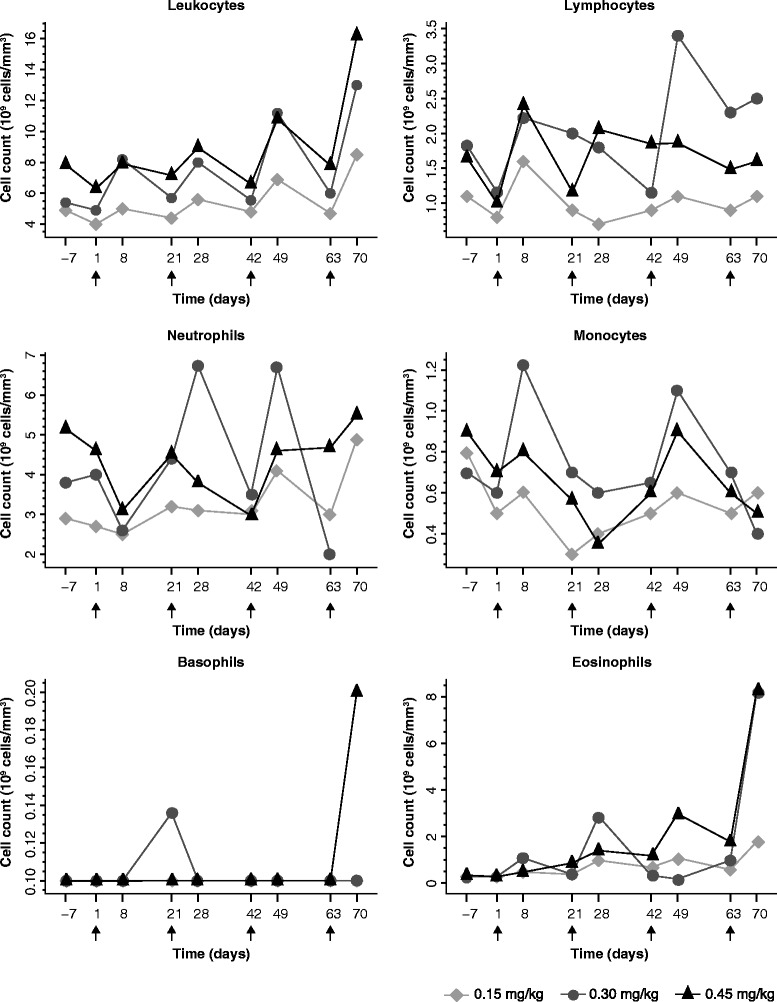
**Time course of leukocyte subpopulations in metastatic NSCLC patients treated with NHS-IL2 (by dose group).** Absolute counts (10^9^ cells/mm^3^) are shown for total leukocytes, lymphocytes, neutrophils, monocytes, basophils, and eosinophils from NSCLC patients treated with NHS-IL2 at dose levels 0.15 mg/kg, 0.30 mg/kg, and 0.45 mg/kg. Geometric mean values of absolute counts are shown for each dose level. Blood was drawn 7 days before start of treatment and at days 1 and 8 of each cycle until discontinuation. The arrow identifies the first of the 3 consecutive days of NHS-IL2 infusion. NSCLC, non-small cell lung cancer.

Blood analysis showed a clear immune induction as defined by the leukocyte kinetics and the production of acute-phase proteins. One week following the start of each cycle (day 1, 21, 42, and 63) an increase of leukocyte counts was noted, followed by normalization with return to basal level (Figure 3). A similar pattern was observed for lymphocytes, monocytes, and neutrophils, with exception of a short depletion of neutrophils during the first cycle. Increase of eosinophils appeared with the second cycle of treatment and showed a similar pattern for all dose levels, but with a highest response after the fourth course. Basophils presented only a modest variation during treatment.

**Figure 4:**
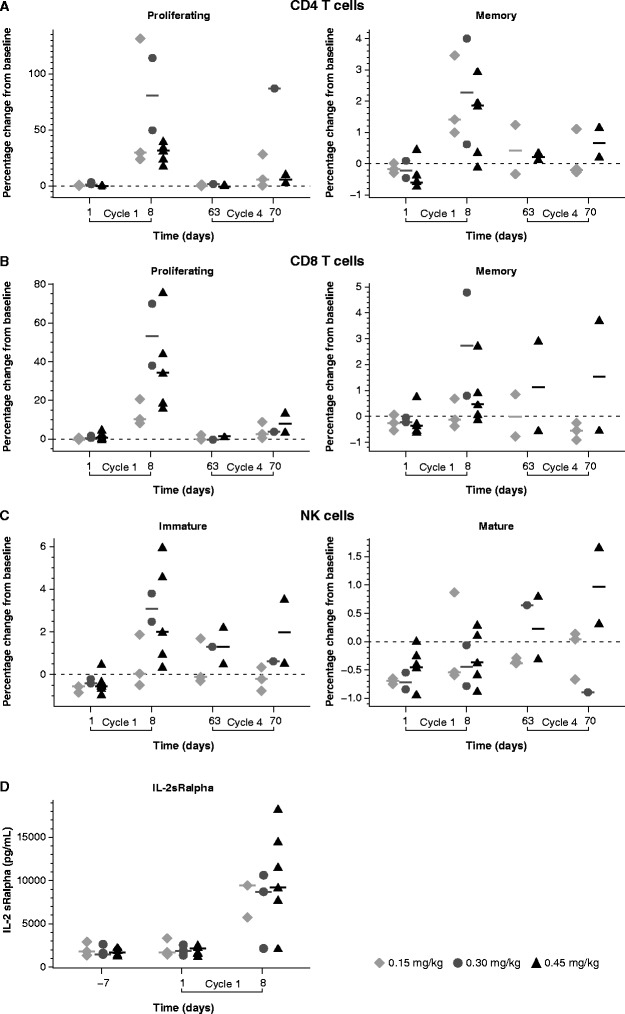
**Time course of lymphocyte subsets (median by treatment group).** Percentage change from baseline (day –7) based on absolute count values (cells/mm^3^) on days 1 and 8 during the first and second treatment cycles for: **(A)** proliferative CD4 T cells (CD3 + CD4 + Ki67+) and memory CD4 T cells (CD3 + CD4 + CD197–CD45RA–); **(B)** proliferative CD8 T cells (CD3 + CD8 + Ki67+) and memory CD8 T cells (CD3 + CD8 + CD197–CD45RA–); and **(C)** immature NK cells (CD16–CD56brightCD3–) and mature NK cells CD16 + CD56 + CD3–. **(D)** sIL-2Ralpha serum concentrations (pg/mL) at baseline and on days 1 and 8 during the first cycle. Graphs show the individual values from 12 NSCLC patients treated with NHS-IL2 at doses of 0.15 mg/kg, 0.30 mg/kg, and 0.45 mg/kg. Bars represent geometric mean values. NK, natural killer; NSCLC, non-small cell lung cancer; sIL-2Ralpha, soluble IL-2 receptor alpha.

In line with the leukocyte kinetic, immunomonitoring showed a considerable increase of proliferating (Ki67 + HLA–DR+) CD4 and CD8 T cells as well as memory (CD197–CD45RA–) CD4 and CD8 T cells during the first cycle of treatment (Figure 4A, B). From the NK population, only immature NK cells (CD16–CD56bright CD3–) increased notably during the first cycle compared to mature (CD16 + CD56 + CD3–) ones (Figure 4C). Analysis of regulatory CD4 + CD25 + Foxp3+ T cells (Tregs) was not performed due to technical issues. In the previous phase I trial in patients with advanced, refractory solid tumors in which NHS-IL2 was used as monotherapy, it was shown that circulating Tregs responded actively to NHS-IL2 with a significant but transitory expansion early after each infusion [18]. The acute-phase proteins sIL-2Ralpha, C-reactive protein, and D-dimer showed a similar cycle-dependent pattern with a strong induction of about 5-fold reaching considerably high levels (>1000 pg/mL) 1 week after infusion (Figure 4D, and data not shown). Of note, increase in sIL-2Ralpha was observed after infusion of NHS-IL2 and not post-irradiation. The concentration of sIL-2Ralpha returned to near baseline level after each cycle. Neopterin and IP-10 (CXCL10) concentrations also increased in response to treatment, although to a lower extent than sIL-2Ralpha (data not shown).

### Clinical outcome

No objective responses were observed during the trial, based on RECIST v1.0 criteria for tumor response, PFS, and OS. Median PFS was 2.9 months (95% confidence interval [CI] 1.5; 3.1) and median OS was 8.6 months (95% CI 4.9; not evaluable). As of the day of reporting there are 2 long-term survivors (both in good performance status 4 years after start of first-line chemotherapy). One patient discontinued treatment with NHS-IL2 in November 2013 and there were still no signs of disease activity in August 2014. Of note, both long-term survivors developed thyroiditis during treatment.

## Discussion

Here we report the first trial in metastatic NSCLC that combined active immunotherapy with the immunocytokine NHS-IL2 plus radiotherapy, with the intention to develop a tumor-specific immune response. Although NSCLC has been seen as a malignancy with low immunogenicity, there is substantial evidence that the immune system plays a central role and that immunotherapy is an appropriate strategy to generate an antitumor immune response [6]. Indeed, several phase I and II trials investigating vaccine-based immunotherapy or immune-checkpoint-pathway inhibitors have shown promise in patients with NSCLC and other solid tumors [3-5]. The rationale for the combination of NHS-IL2 and radiotherapy was to modulate the immunologic microenvironment of the tumor by local irradiation and thereby enhance the IL-2–mediated effects of NHS-IL2. Indeed, in addition to the direct antitumor effects of radiation, such as genetic damage and enhanced cross-presentation of tumor antigens, local irradiation can also affect immune regulatory pathways, leading to an enhanced immune active antitumor response [25]. This is illustrated by experiments in a poorly immunogenic mouse carcinoma, in which ionizing radiation induced upregulation of chemokine CXCL16, which recruited effector CD8+ T cells to the tumor [26]. Building on the investigational concept that radiation treatment increases the immunogenicity of otherwise poorly immunogenic tumors, our preclinical data showing efficacy in the weakly immunogenic LLC model supports the notion that radiation can enhance the efficacy of immunotherapy. We tested the same NHS-IL2 plus chemo-radiotherapy combination in the 4T1 breast cancer and CT26 colon cancer models and saw no enhancement of the chemo-radiation effect. Whether the lack of a combination effect was due to an induction of Treg in these models was not fully explored nor has a thorough investigation of suppressor populations been performed in the current preclinical study.

The combination of NHS-IL2 with local irradiation showed an enhanced antitumor effect in the LLC animal model. These preclinical results led to a multicenter phase Ib trial to test the combination of NHS-IL2 and local radiation therapy in patients with metastatic NSCLC. Platinum-based therapy, which is the standard of care in metastatic NSCLC, was given prior to the combination of NHS-IL2 and radiation to allow for leukocyte/lymphocyte recovery post nadir [27]. The combination of irradiation followed by NHS-IL2 after first-line chemotherapy in metastatic NSCLC patients was well tolerated. The treatment in a small patient cohort showed an acceptable safety profile and the MTD was not reached, as the highest dose tested in these patients was 0.45 mg/kg. Data from the phase I study in solid tumors confirmed that the MTD of NHS-IL2 monotherapy was reached at 0.6 mg/kg [11]. Side effects were manageable and were mostly related to influenza-like symptoms and rash. It is likely that the skin-related AEs observed in this trial have the same etiology as the toxicity related to systemic IL-2, which is erythematous rash resulting from the perivascular infiltration of T cells. However, in this trial, following administration of NHS-IL2, the skin rash was both mild and self-limiting. In contrast to normal recombinant IL-2, no severe cardiovascular events such as hypotension or vascular leak syndrome were observed. Mild and transient facial swelling and peripheral edema in 3 out of 13 patients were the only signs of fluid extravasation, suggesting a better tolerability of NHS-IL2 compared to IL-2 therapy.

Pharmacokinetic analysis revealed no drug accumulation with repeated exposure; the slightly higher post-dose concentrations in cycle 4 (week 10) observed for the 0.15 and 0.30 mg/kg doses might be explained by variability in the measurements and a small sample size.

A recent study reported on large numbers of viable circulating tumor cells during radiotherapy in NSCLC patients [28]. These tumor cells and the corresponding immune response might be responsible for the abscopal effect. Herein, several lines of evidence suggest that NHS-IL2 can initiate an immune response. First, both leukocyte kinetics and FACS analysis data indicate a cycle-dependent induction of a general type of immune response, similar to that recently described [18]. This immune response was not restricted to lymphocytes: a pronounced cycle-dependent increase in both neutrophil and eosinophil number was also observed. A more detailed analysis of immune response (CD4, CD8, Treg profile including activation/proliferation stage) both in blood and in tumor biopsies is planned to be re-investigated in the phase II trial in metastatic melanoma, in which NHS-IL2 is given with stereotactic body irradiation (clinicaltrial.gov identifier NCT01973608). The biopsy investigation may help shed light on human leukocyte antigen (HLA) expression as a measure of how tumors respond to various treatments based on underlying immunogenicity [29]. The decrease of the immune response at cycle 4 compared to cycle 1 observed in this trial has also been observed previously in a phase I trial in solid tumors [18]. The hypothesis of possible T-cell re-distribution (blood versus tumor) will be investigated in the ongoing phase II trial. Second, the thyroid incidents were shown in 3 out of 13 patients, while this has not been observed in the phase I trial on NHS-IL2 single-agent therapy in solid tumors [11]. In the current phase Ib trial, transient hyperthyroidism was observed in 3 patients, and in 2 out of 3 patients this was followed by permanent hypothyroidism requiring thyroid hormone replacement treatment. No other endocrinopathies were observed, suggesting either a tissue specificity of the immune response or a relative higher vulnerability of the thyroid gland. The net result was a loss of thyroid function in 2 patients and temporary increased function in the other. However, the latter patient also showed rapid progressive disease. The mechanism underlying these incidents remains obscure, as no autoantibodies were detected and no biopsies were taken. Most likely a lymphocyte-mediated inflammation resulted in the impairment of thyroid function.

The unexpectedly long-term disease control in 1 out of 13 patients is compatible with the possibility of antitumor response. However, no direct evidence was generated to support this hypothesis. A number of other preclinical and clinical studies exploited the pro-immunogenic effects of local radiotherapy that promote the effector phase of tumor rejection, by combining radiation with immune therapy [12,30,31]. A recent case report suggests that the success of the combination of local radiotherapy and anti–CTL-associated antigen 4 can be observed in melanoma patients [13]. Together, these data and the results from recent clinical trials with the anti-programmed death-1 (aPD-1) and anti-programmed death ligand-1 (aPD-L1) monoclonal antibodies suggest that the process of tumor escape to immunotherapy can be reverted [4,19,32].

## Conclusions

It is feasible to combine current anticancer treatment modalities with immunotherapy. NHS-IL2 preceded by local irradiation revealed tumor growth inhibition in the LLC tumor model. The phase Ib trial provides evidence that the combination of local radiotherapy with NHS-IL2 is well tolerated, and the limited antitumor activity data suggest that a small cohort of NSCLC patients might benefit from this treatment. These findings support further studies combining radiation with NHS-IL12 in patients with solid tumors.

## Additional file

Additional file 1: Figure S1. Gene expression in tumors following treatment with radiotherapy and/or NHS-IL2 on days 10 and 13. Four representative tumors from each group were chosen for analysis. Gene expression was measured by quantitative PCR. FASL, Fas ligand; FOXP3, forkhead box protein 3; GZMB, granzyme B; PBS, phosphate buffered saline; PCR, polymerase chain reaction; PRF1, perforin 1; RTX, radiotherapy.

## Abbreviations

AEs: Adverse events
ANOVA: Analysis of variance
APC: Allophycocyanin
CI: Confidence interval
CTLs: Cytotoxic T-lymphocytes
DMEM: Dulbecco’s modified eagle’s medium
ECOG: Eastern cooperative oncology group
EGFR: Epidermal growth factor receptor
ELISA: Enzyme-linked immunosorbent assay
FACS: Fluorescence-activated cell sorting
FITC: Fluorescein isothiocyanate 1
HLA-DR: Human leukocyte antigen D-related
IHC: Immunohistochemistry
IL-2: Interleukin-2
IP: Interferon-gamma–induced protein
i.v.: Intravenously
LLC: Lewis lung carcinoma
mAb: Monoclonal antibody
MTD: Maximum tolerated dose
NCI CTCAE: National cancer institute common terminology criteria for adverse events
NK: Natural killer
NSCLC: Non-small cell lung cancer
OS: Overall survival
PCR: Polymerase chain reaction
PD-1: Programmed death-1
PD-L1: Programmed death ligand-1
PE: Phycoerythrin
PFS: Progression-free survival
RECIST: Response evaluation criteria in solid tumors
TKI: Tyrosine kinase inhibitor
TMB: 3,3′5,5′-tetramethylbenzidine
Tregs: Regulatory CD4 + CD25 + Foxp3+ T cells
