# Supplementary figures and images for: NHS-IL2 combined with radiotherapy: preclinical rationale and phase Ib trial results in metastatic non-small cell lung cancer following first-line chemotherapy
Source: J Transl Med. 2015 Jan 27;13:32. doi: 10.1186/s12967-015-0397-0 (PMC4320467; doi:10.1186/s12967-015-0397-0)

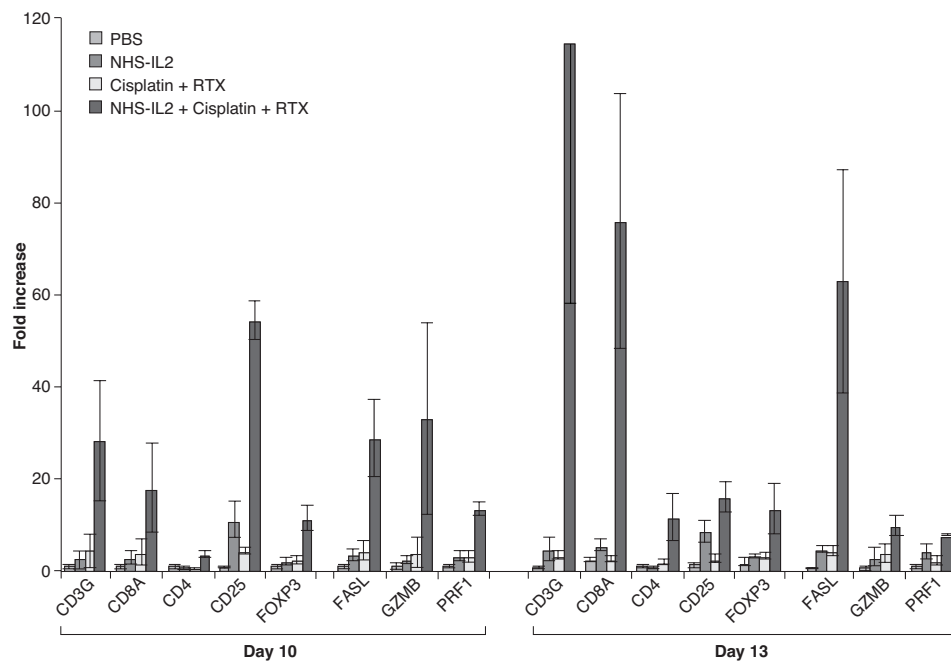

Supplement: Additional file 1: Figure S1. — Gene expression in tumors following treatment with radiotherapy and/or NHS-IL2 on days 10 and 13. Four representative tumors from each group were chosen for analysis. Gene expression was measured by quantitative PCR. FASL, Fas ligand; FOXP3, forkhead box protein 3; GZMB, granzyme B; PBS, phosphate buffered saline; PCR, polymerase chain reaction; PRF1, perforin 1; RTX, radiotherapy. [file 12967_2015_397_MOESM1_ESM.pdf]
